# Brief health literacy assessment as an enhancement to existing cognitive screening tests: an exploratory investigation

**DOI:** 10.1002/alz.089845

**Published:** 2025-01-03

**Authors:** Abigail Vogeley, Laura M Curtis, Lauren Opsasnick, Stephanie Batio, Andrea Russell, Sophia W. Light, Fangyu Yeh, Julia Yoshino‐Benavente, Morgan Bonham, Rebecca Lovett, Michael S Wolf

**Affiliations:** ^1^ Northwestern University, Chicago, IL USA; ^2^ Northwestern University Feinberg School of Medicine, Chicago, IL USA

## Abstract

**Background:**

Most cognitive screening tests used in primary care to identify adults with cognitive impairment, including Alzheimer’s disease and related dementias, have been limited in their diagnostic accuracy, especially in mild cases. Resultant false positives or false negatives (e.g., delayed diagnoses) have respective harms. While expressed concern of an individual’s real‐world self‐care abilities from a caregiver can improve confidence in a screening result, as many as half of patients with cognitive complaints may lack this informant. We explored whether the addition of an objective, brief, health literacy assessment could improve the accuracy of a longstanding, common cognitive screening tool.

**Method:**

Data came from a longitudinal aging study of older adults from Chicago, IL. Cognitive function was assessed using Z‐scores generated from a comprehensive neurocognitive battery of 13 measured across 5 domains. Participants were determined to have an impairment if they scored ←1 on ≥2 tests within at least one domain. The Mini Mental Status Exam (MMSE) and the Newest Vital Sign (NVS), a common 3‐minute, 6‐item measure of health literacy, were administered. Receiver operating characteristic (ROC) curves assessed the specificity and sensitivity of adjusted MMSE models (cut‐off score ≤26) with and without the NVS to detect cognitive impairment. Areas under the curve (AUCs) were compared using the DeLong test.

**Result:**

Participants (N = 389) were on average 71 years old (SD 5.2); most participants were female (72%) and non‐Hispanic White (56%). Most (86%) had 2+ chronic conditions (M 3.4 (SD 1.8)), and 28% had at least a mild cognitive impairment based on our comprehensive neurocognitive battery. ROC analyses indicated that cognitive impairment detection by the MMSE alone (AUC = 0.79, 95% CI [0.73‐0.84]) was significantly improved (Z = ‐3.68, 95% CI [0.03,0.11], p‐value = <0.001) with inclusion of the NVS (AUC = 0.86, 95% CI [0.82‐0.90]).

**Conclusion:**

Our findings demonstrate the potential benefit of integrating a brief assessment of real‐world self‐care task performance and health literacy, such as the NVS, with routine cognitive screening tests to improve accurate identification of cognitive impairment in primary care. Improving the accuracy of common screening tools may help facilitate earlier detection, diagnosis, and subsequent care.

